# Quantitative Functional Assessment of Patients Undergoing En Bloc Vertebrectomy for Spinal Tumors: A Multi-Segment Motion Analysis Study

**DOI:** 10.3390/cancers18162599

**Published:** 2026-08-12

**Authors:** Francesca Bressan, Riccardo Ghermandi, Cristiana Griffoni, Andrea Giacovazzo, Riccardo Rosa, Valerio Pipola, Chiara Cini, Bruna Maccaferri, Lisa Berti, Alberto Leardini, Alessandro Gasbarrini

**Affiliations:** 1 Movement Analysis Laboratory IRCCS Istituto Ortopedico Rizzoli, 40136 Bologna, Italy; francesca.bressan@ior.it (F.B.); leardini@ior.it (A.L.); 2 Complex Spinal Surgery Unit, IRCCS Istituto Ortopedico Rizzoli, 40136 Bologna, Italy; riccardo.ghermandi@ior.it (R.G.); cristiana.griffoni@ior.it (C.G.); valerio.pipola@ior.it (V.P.); chiara.cini@ior.it (C.C.); 3 Physical Medicine and Rehabilitation Unit, IRCCS Istituto Ortopedico Rizzoli, 40136 Bologna, Italy; andrea.giacovazzo@ior.it (A.G.); riccardo.rosa@ior.it (R.R.); lisa.berti@ior.it (L.B.); 4 Department of Biomedical and Neuromotor Sciences, Alma Mater Studiorum, University of Bologna, 40127 Bologna, Italy

**Keywords:** 3D gait analysis, multi-segment spine model, trunk kinematics, spinal tumors, en bloc vertebrectomy

## Abstract

**Objectives**: Spinal tumors often require complex surgical treatments, in particular en bloc vertebrectomy, and a pre-operative kinematics and functional assessment of the spine could be advised to improve surgical outcomes and patients’ quality of life. However, established protocols for stereophotogrammetry-based gait analysis are not available. This study aims at evaluating the feasibility of an experimental protocol for multi-segment trunk and spine kinematics in patients scheduled for en bloc vertebrectomy. **Methods**: Ten patients and ten age- and sex-matched healthy controls underwent full-body gait analysis using an established marker-set able to track the lower limbs, pelvis, thorax, shoulders, and five spine line segments. The participants completed static double leg standing, barefoot level walking, and elementary trunk movements. A preliminary within-session repeatability assessment in two individuals (one representative patient and one healthy participant) was performed (root mean square error smaller than 10% of ROM; intra-class correlation coefficients > 0.8). **Results**: Compared with controls, patients affected by spinal tumors showed slower gait, shorter stride length, smaller ROM at the pelvis and thorax, and reduced frontal-plane mobility between spine line segments, but physiological lower-limb joint kinematics. During elementary movements, patients exhibited reduced trunk ROM in the anatomical plane of motion, particularly at a central spine segment, and smaller pelvic mobility in axial rotations. **Conclusions**: The results of this study support the feasibility of the proposed protocol for gait analysis in the rare clinical population of patients affected by spinal tumors and provide descriptive functional information that may complement conventional pre-operative clinical and imaging assessments.

## Simple Summary

So-called en bloc vertebrectomy is a challenging surgical procedure aimed at removing one or more vertebral segments which are affected by a spinal tumor, while preserving or restoring spinal stability, neurological function, and quality of life. Unfortunately, conventional medical imaging cannot quantify the impact of the tumor on the patient’s movement and daily living functionality. The present study employed an advanced 3D motion analysis protocol to quantify the movement of the lower limbs, spine, and trunk during walking and elementary trunk exercises in patients scheduled for en bloc vertebrectomy. The results of this study suggest that patients eligible for this surgical intervention exhibit specific movement limitations, including reduced walking speed and diminished spinal mobility. These analyses provide a reference for pre-operative functional assessments, in addition to conventional radiographic imaging.

## 1. Introduction

In recent years, spinal surgery has undergone significant advancements, with the development of increasingly sophisticated techniques aimed at improving clinical and functional outcomes. Among these techniques, surgical approaches for spinal oncology, particularly en bloc resection, have gained prominence in the management of primary and metastatic vertebral tumors [1,2,3]. En bloc resection involves the complete removal of one or more vertebral segments affected by the tumor and is aimed at achieving local disease control while preserving or restoring spinal stability and neurological function [1]. Still, these bone tumors and clinical conditions are very rare; vertebrectomy is indicated only in selected cases, being a challenging and demanding surgical procedure performed only in a few highly specialized spinal surgery centers. Thus, the population size in relevant studies is usually small.

The development of new surgical techniques aiming at minimally invasive approaches also requires more comprehensive functional parameters to improve the patient’s postoperative recovery and the quality of life. Conventional tools such as imaging and clinical scales (e.g., Oswestry Disability Index, Visual Analog Scale) do not always provide a comprehensive picture of functional recovery, particularly in terms of locomotor capacity.

In this context, three-dimensional gait analysis emerges as a valuable objective tool for quantifying functional status and for detecting alterations in locomotion [4]. Gait analysis is a powerful, non-invasive, and objective tool used to evaluate various orthopedic, neurological, and neuromuscular conditions. By measuring parameters such as velocity, step length, cadence, joint angles, and muscle activation (EMG), it allows clinicians to accurately quantify the severity of functional impairments, and to distinguish between primary conditions and compensatory mechanisms. Through the measurement of spatiotemporal parameters and kinematic and kinetic patterns, gait analysis allows clinicians to assess how spinal pathology, and then case-relevant surgical treatments, can alter walking ability, expressed, e.g., through progress speed, balance, and neuromuscular control [5,6,7,8]. These analyses can be particularly useful in cases of complex oncological surgical treatments, such as vertebrectomy, to support surgical planning from a functional perspective. Our center has considerable experience in the treatment of spinal tumors, but the impact of these pathologies and their surgical treatments on the overall mobility of the spine has not yet been investigated in the literature. As the aim of this surgical treatment is the removal of the tumor according to oncologic principles [9], the spine is usually analyzed before and after surgery using diagnostic imaging in the static upright condition (CT scan, MRI, X-ray). In patients with spinal malignancies, a correlation was found between spino-pelvic alignment from whole-spine lateral radiographs before and after en bloc resection and patient-reported outcomes [10]. The present paper analyzes locomotion alterations, with particular attention to trunk mobility, in patients undergoing en bloc resection of one or more vertebrae for spinal tumors.

Stereophotogrammetric motion analysis is extensively used for the assessment of body segment kinematics during gait and other functional tasks [11]. For the study of lower-limb body segments and joints, definitions are quite consistent among experimental protocols, while for trunk and spine kinematics, a standard multi-segmental approach is still lacking. Spine motion is very complex, and the overall motion occurs as a combination of three-dimensional rotations at many different small joints, i.e., intervertebral disks. In addition, non-invasive tracking of individual vertebrae in three dimensions is very difficult. Despite a large variety of proposals, no unified or standardized protocol to assess spine motion has been established yet. Existing protocols can be classified according to the anatomical regions analyzed, namely, the entire trunk, the thorax, the spine, and the shoulder girdle, as well as the upper trunk, the lower trunk, and the lumbar spine. Leardini et al. [12] quantitatively compared the results of eight different protocols used to evaluate trunk kinematics during locomotion, demonstrating large differences in the pattern and the range of motion (ROM).

Pioneering experimental protocols for trunk and spine motion [13] assumed the overall trunk as a single rigid body; given that the cervical, thoracic, and lumbar spine and the thorax exhibit distinct movements, these protocols were found to be inadequate. Subsequent studies have employed multi-segment models, which incorporate multiple spinal segments, including the thoracic spine and cage, the lumbar spine, and the shoulder girdle [11,14], even across different activities [15]. Both review papers [11,14] have revealed heterogeneity in marker placement and spinal segment definitions and have highlighted the absence of standardized protocols; the former also claimed that of the 74 papers reviewed, only 35% of the proposed methods were suitable for clinical applications. A study by Leardini et al. [15] proposed an experimental protocol for trunk motion in which the spine was divided into five-line segments, the thorax was tracked in three dimensions, and the shoulder was tracked as a line segment. This apparently represents a satisfactory compromise between a resolution higher than that of pioneering protocols and the necessary simplicity for feasible experiments in clinical gait analysis. High intra-subject repeatability and inter-subject variability were also demonstrated.

None of these studies have applied stereophotogrammetric motion analysis to patients with spinal tumors. Previous research has primarily focused on other spinal pathologies—such as scoliosis, spinal adult deformities, low back pain, or post-fusion conditions; however, this demonstrates the feasibility and clinical utility of multi-segmental spine models [16,17,18,19,20]. A state-of-the-art gait analysis method involving the trunk is implemented here in a population of patients that will be subjected to en bloc resection for spinal tumors. Kinematic analyses are performed to compare these patients with a similar population of healthy subjects, in static upright posture but also during the execution of level walking at a natural self-selected speed and of elementary trunk movements.

The presented original analyses assess the feasibility of trunk motion measurements in this patient population, providing a reference for pre-operative kinematics and functional assessment, which could support surgical planning and clinical outcomes.

## 2. Materials and Methods

### 2.1. Study Design and Population

This prospective observational study included adult patients diagnosed with primary or metastatic spinal tumors who were scheduled for en bloc vertebrectomy. Ten patients (Table 1) were consecutively recruited at the IRCCS Istituto Ortopedico Rizzoli (Bologna, Italy), after obtaining written informed consent. The study protocol was approved by the Institutional Ethics Committee CE AVEC (n° 151/2023/Sper/IOR, 16 February 2023). Inclusion criteria were (i) age ≥ 18 years, (ii) surgical indication for vertebrectomy at cervical, thoracic, or lumbar levels, and (iii) ability to walk independently prior to surgery and after six months. Exclusion criteria included severe neuromotor deficits, systemic conditions affecting gait, and inability to walk independently at six-month follow-up. Patients were subdivided into two groups (Table 1) based on tumor location and thus vertebrectomy level: thoracic and lumbar.

A control group of ten asymptomatic adult subjects, matched for age, sex, and BMI distribution, was also recruited to provide normative reference data. Control subjects (3 females and 7 males with a mean age of 45 ± 15.7 years and a mean BMI 24.9 ± 3.2) had no history of spinal or lower-limb surgery, no musculoskeletal disorders, and no pain or neurological conditions affecting gait.

### 2.2. Study Experimental Protocol and Analyses

**Figure 1:**
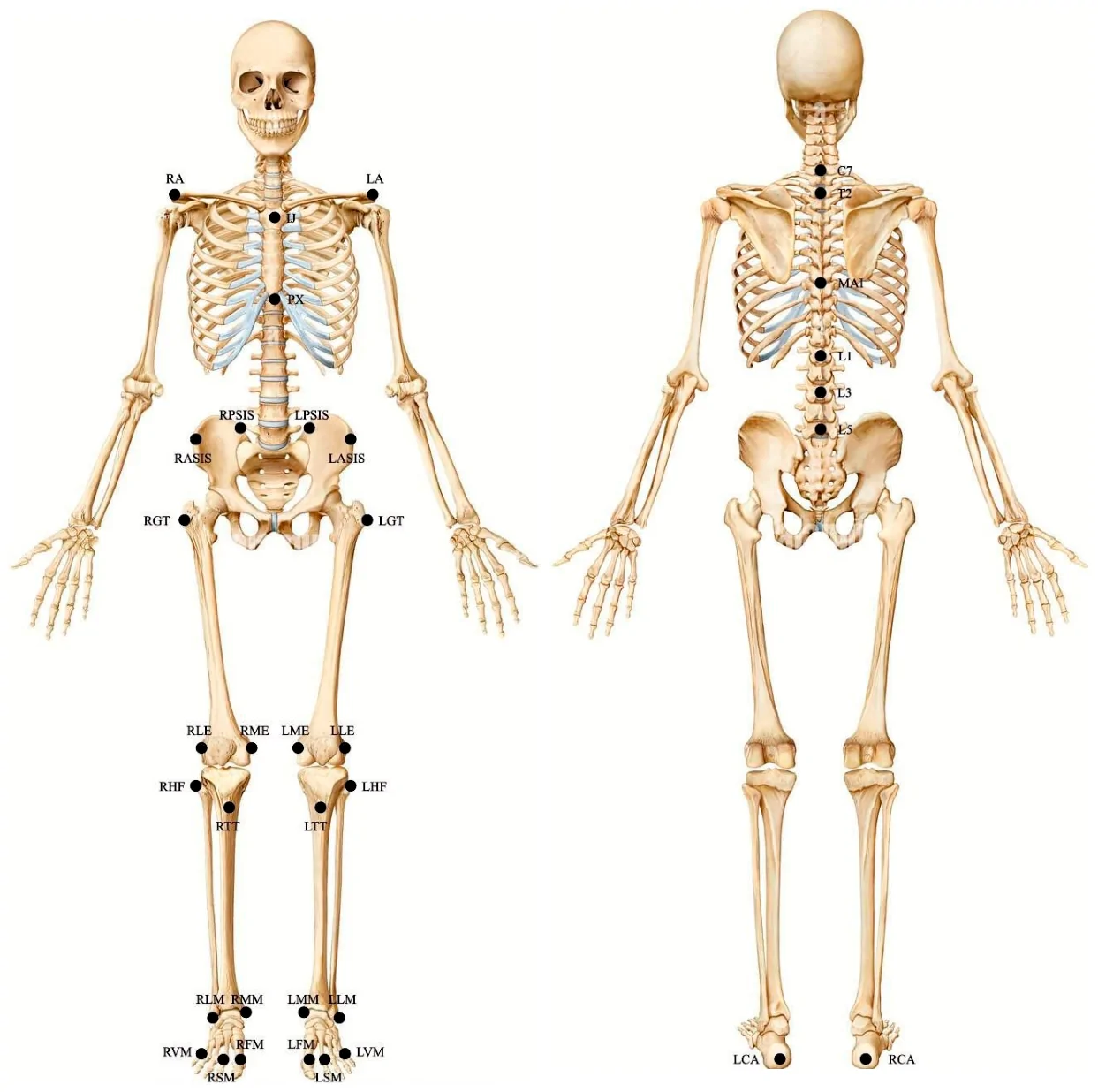
Views from the front (left) and from the back (right) of the marker-set: greater trochanter (RGT, LGT); medial epicondyles (RME, LME); lateral epicondyles (RLE, LLE); tibial tuberosity (RTT, LTT); head of the fibula (RHF, LHF); medial malleoli (RMM, LMM); lateral malleoli (RLM, LLM); calcaneus (RCA, LCA); first, second, and fifth metatarsals (RFM, RSM, RVM, LFM, LSM, LVM); right and left anterior superior iliac spines (RASIS, LASIS); right and left posterior superior iliac spines (RPSIS, LPSIS); jugular notch (IJ); xiphoid process (PX); right and left acromion (RA, LA); midpoint of the scapular apices (MAI); spinous processes of the seventh cervical vertebra (C7); second thoracic vertebra (T2); and first, third, and fifth lumbar vertebrae (L1, L3, L5).

A 3D motion capture system (Vicon 612, Vicon Motion Capture, Oxford, UK) with nine infrared cameras operating at 100 Hz was used to record full-body kinematics during barefoot gait of the patients prior to surgery and of the control group. All subjects were instrumented with 37 spherical reflective markers (14 mm diameter) in accordance with a state-of-the-art experimental technique. The protocol combined the IORGait protocol for the lower limbs [21], and a subsequent protocol for the trunk and spine [15] (Figure 1). The marker placement reliability was evaluated between two different examiners. A static double-leg standing weight-bearing posture (300 frames) and five level walking trials at a self-selected comfortable speed were recorded. Participants also performed one trial consisting of three repetitions for each of the following elementary movements of the entire trunk: flexion–extension, lateral bending (on both sides), and axial rotation (in both directions). The participants were instructed to refrain from making any compensatory movements, such as lower-limb joint rotations.

The labeling of the markers and 3D reconstruction and interpolation of the marker trajectories were performed using Vicon standard software tools. Marker trajectories were low-pass-filtered using a fourth-order zero-lag Butterworth filter. For walking trials, cycle identification was obtained by a single experienced operator according to standard procedures using force-plate contacts and foot-marker trajectories. In the three elementary movements, the cycles were manually identified using standard stick diagram animations, by observing the onset and cessation of a relevant trunk marker. Time-histories of joint kinematics were normalized with respect to gait cycle duration to facilitate the comparison of curves.

All acquisitions were processed in Python according to the protocols. The following spatiotemporal gait parameters were obtained from foot markers’ trajectories and of the ground reaction force vector: stance time, standard and normalized stride lengths, and standard and normalized walking speeds. For the kinematic measurements in all dynamic acquisitions, the corresponding value in static acquisitions, i.e., offsets, was subtracted. Rotations were calculated between adjacent segments, apart from the thorax, where 3D motion relative to both the pelvic and laboratory reference frames was tracked. Between line segments, planar rotations were obtained, projecting relevant markers on the segment anatomical frames, of the thorax for the shoulder line segment, and on the pelvis for the spine line segments. The lumbo-pelvic ratio (LPR) was measured as the ratio between ROM in the sagittal plane of the pelvis and of the lumbar spine, the latter being the sum of the planar rotations of its two-line segments.

For each kinematic measurement, the 50th (median), 75th, and 25th percentiles were computed, and represented in the time-history by a central median curve and a 25-to-75th percentile band. Intra-subject repeatability of the measurements was assessed in one representative patient (TS0) and one healthy subject by calculating the root mean square error (RMSE) and the intra-class correlation coefficient (ICC) across the walking trials and repetitions. For the elementary trunk movements, only the RMSE was calculated, because of the limited number of repetitions available (n = 3). Furthermore, inter-subject variability and reliability of the measurements were evaluated separately in the pathological and control groups using a two-way mixed-effects intra-class correlation coefficient with absolute agreement and average measures (ICC(3,k)). Ninety-five percent confidence intervals (95% CI) were computed. Absolute reliability was additionally quantified through the standard error of measurement (SEM). Given the limited sample size and the marked clinical heterogeneity of the patient cohort, no inferential statistical comparisons between patients and controls were performed. The analyses were therefore descriptive and aimed at characterizing movement patterns rather than testing predefined hypotheses.

### 2.3. Patient-Reported Outcomes

Before the biomechanical assessment, patient-reported outcomes (PROMs) were collected for each patient to measure pain (VAS score), functional ability (Oswestry Disability Index, ODI) and the fear of falling, self-efficacy, and balance confidence (FES-I).

## 3. Results

### 3.1. Repeatability

#### 3.1.1. Intra-Subject Repeatability

The study protocol demonstrated high repeatability during both level walking and elementary trunk movements in the asymptomatic subject and the patient.

During level walking, lower-limb joint rotations showed excellent consistency (RMSE < 1.3°, ICC > 0.94 in the asymptomatic subject; RMSE < 1.8°, ICC > 0.78 in the patient). The pelvic RMSE remained below 1.4° (ICC > 0.85) in the asymptomatic subject and below 0.9° (ICC > 0.70) in the patient, while thoracic rotations showed very low variability (RMSE 0.2–0.7°, ICC > 0.86). Spinal and shoulder segment RMSE ranged from 0.2° to 1.5°, consistently <10% of the corresponding ROM.

For trunk movements, pelvic RMSE was smaller than 5.6° in flexion–extension, 3.2° in lateral bending, and 2.5° in internal–external rotation. The thoracic RMSE ranged from 1.3° to 8.5° depending on the task. Across all movements, the spinal segment RMSE ranged from 0.5° to 2.7°, always remaining below 10% of the ROM. As the repeatability analysis was based on only one representative patient and one healthy participant, it represents a preliminary within-session repeatability assessment and does not establish repeatability across the entire patient and control cohorts.

#### 3.1.2. Inter-Subject Repeatability

The inter-subject reliability analysis showed overall good to excellent reliability for most kinematic variables in both control subjects and patients (Supplementary Material Table S1A–D).

In the control group, the ICC (3, k) values ranged from 0.60 to 0.99, with the majority of variables showing excellent reliability (ICC > 0.90), particularly for thoracic and lumbar lateral bending and flexion. Lower ICC values were observed for thoracic rotation relative to the laboratory reference frame (ICC as low as 0.60).

Similarly, in the patient group, ICC values ranged from 0.50 to 0.99, indicating overall good to excellent reliability across most variables. Excellent reliability was observed for several lumbar and thoracic lateral bending variables (ICC up to 0.99), whereas thoracic rotation relative to the laboratory reference frame showed lower reliability (ICC = 0.50). SEM values were generally low across variables, indicating limited absolute measurement error.

### 3.2. Level Walking

#### 3.2.1. Spatiotemporal Parameters

The spatiotemporal parameters for the patient population are reported in Table 2. The longer stance phase duration in the patients, i.e., 62.3% versus 60.6%, may reflect a more cautious transition to single-limb support. These differences, particularly the markedly slower walking speed, highlight the severity of the patients’ clinical condition.

#### 3.2.2. Lower Limbs and Pelvis

**Figure 2:**
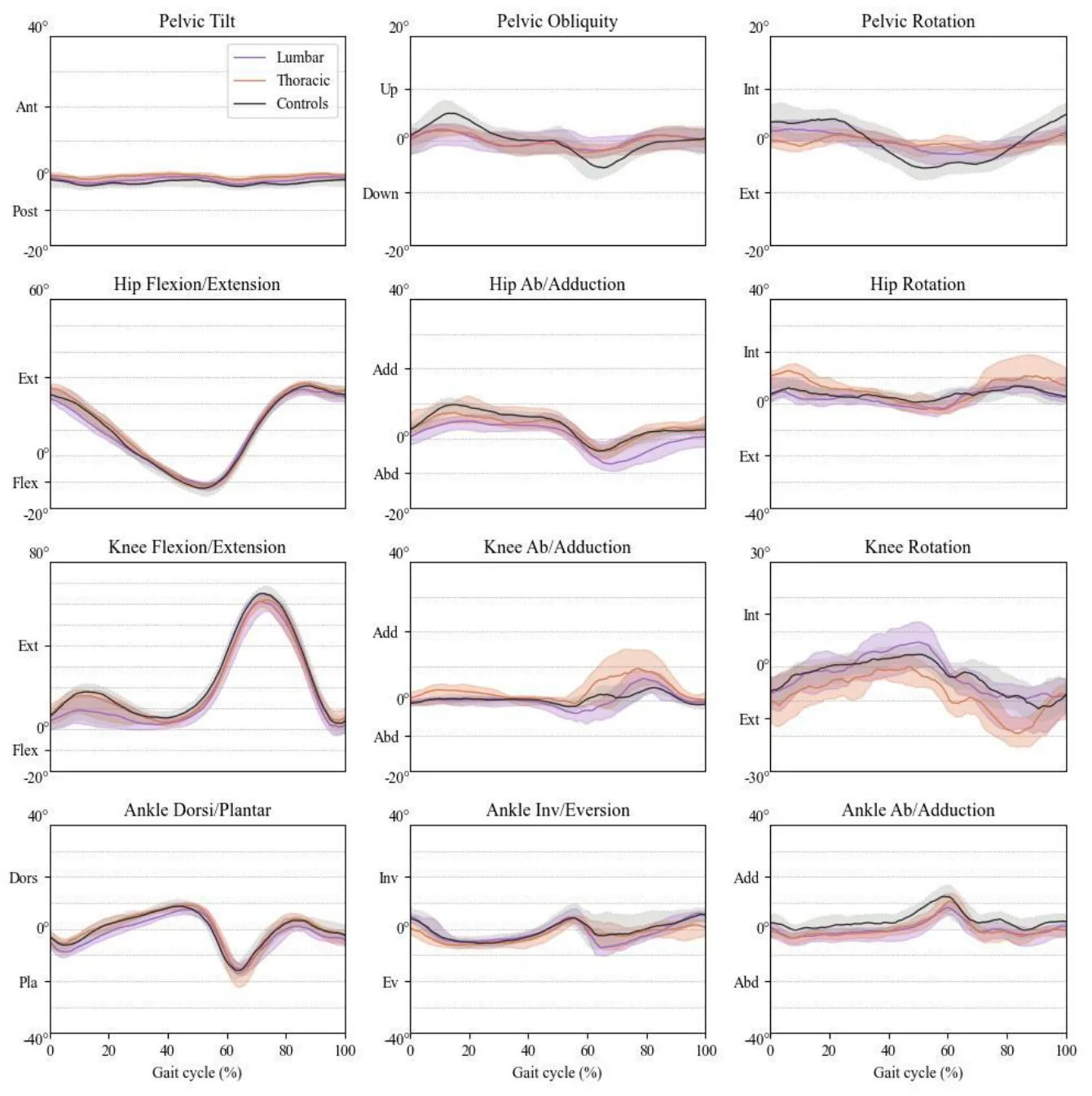
Kinematic curves during gait (normalized cycle, 0 – 100) in the three anatomical planes (sagittal—left, frontal—central, and transverse—right). Rotations of the pelvis (top row) and of the hip, knee, and ankle (following three rows) joints are shown. The plots display the median and 25th–75th percentile of segment and joint rotations for the three groups: lumbar (purple), thoracic (orange), and control (gray).

Physiological rotations were observed for the hip, knee, and ankle joints for both patient groups, as shown in the relevant time-histories (Figure 2). This applies also to the tilt motion (in the laboratory reference frame) of the pelvis, where the range of obliquity and axial rotation appeared smaller than those of the healthy controls.

#### 3.2.3. Thorax and Shoulders

**Figure 3:**
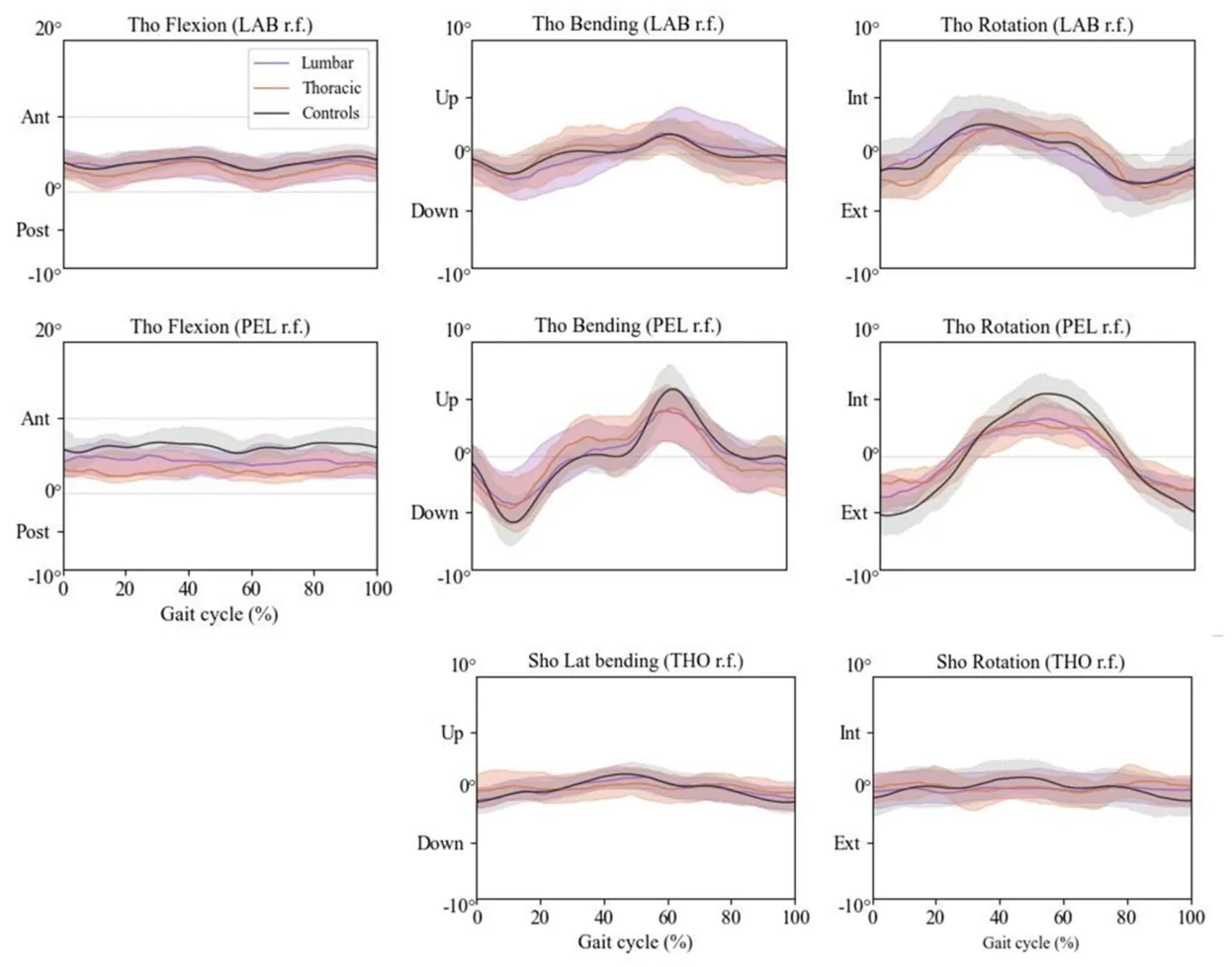
Kinematic curves during gait (normalized cycle, 0–100) in the three anatomical planes (sagittal—left, frontal—central, and transverse—right). Rotations of the thorax (Tho) with respect to the laboratory (LAB—top row) and pelvis (PEL—second row) reference frames, and of the shoulders (Sho) with respect to the thorax (bottom row) are shown. The plots display the median and 25th–75th percentile of segment and joint rotations for the three groups: lumbar (purple), thoracic (orange), and control (gray).

The three-dimensional motion of the thorax in the laboratory frame and of the shoulder line in the thorax frame in the patient population was very similar to that in the control population (Figure 3). Thorax flexion in the pelvis frame appeared smaller in the patient than in the control population. In the frontal, i.e., bending, and transverse planes, i.e., axial rotation, the patterns of motion were similar between the two populations, but the ROM was smaller for the patients, at 8.2 ° vs. 12.2° and 6.9° vs. 11.1°, respectively.

#### 3.2.4. Spine Line Segments

The ROM of the spine segments was similar between the two populations in the sagittal plane and different in the frontal plane (Table 3).

### 3.3. Elementary Movements: Flexion and Extension

For the three elementary trunk movements, only the corresponding rotations in the anatomical plane of the primary motion are reported. Rotations in the other planes were negligible, with ranges of motion below 8°. These small values are consistent with those previously reported by Leardini et al. [15].

**Figure 4:**
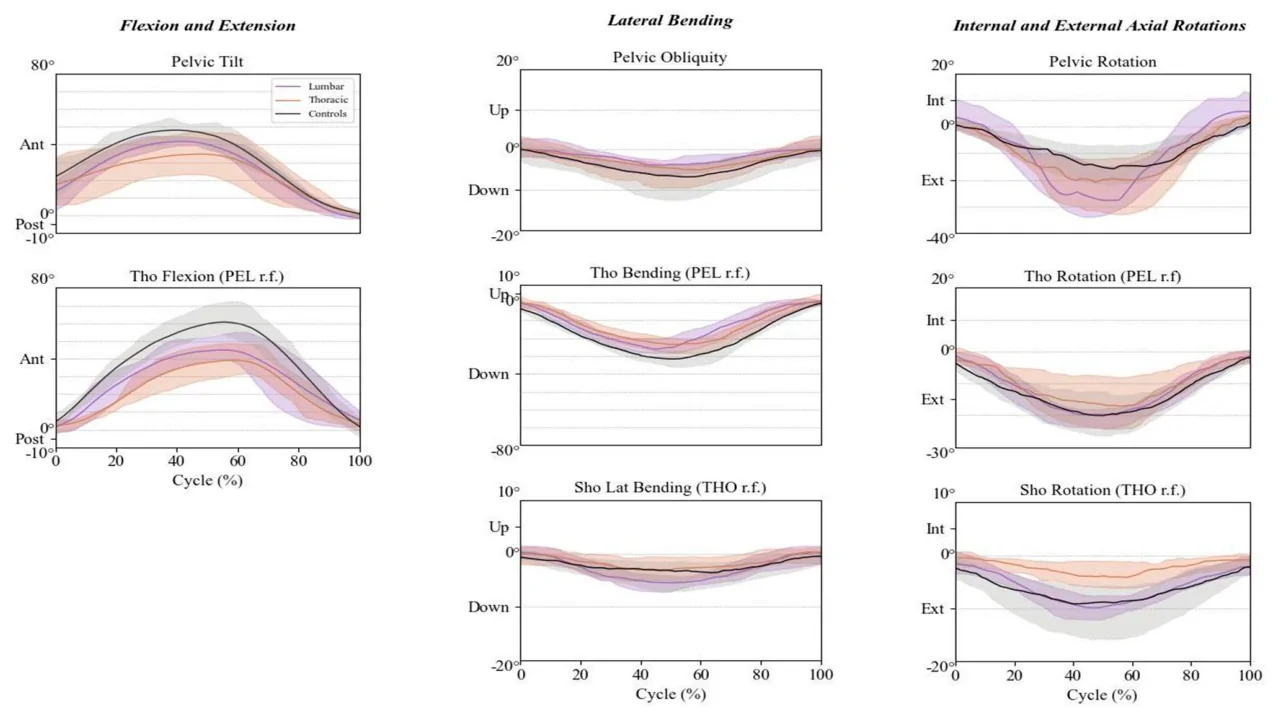
Kinematic curves during the elementary movements (normalized 0–100) in the corresponding anatomical plane. Rotations of the pelvis (top row), of the thorax (Tho) with respect to pelvis (second row) reference frames, and of the shoulders (Sho) with respect to the thorax (bottom row) are shown. The plots display the median and 25th–75th percentile of segment and joint rotations for the three groups: lumbar (purple), thoracic (orange), and control (gray).

During elementary flexion and extension (Figure 4, left column), the pelvic tilt and thorax flexion in the pelvis frame exhibited a smaller ROM in the patients than in the control populations, particularly in the thoracic group, where the ROM was 38.4° vs. 49.1° and 40.8° vs. 58.3°, respectively.

**Figure 5:**
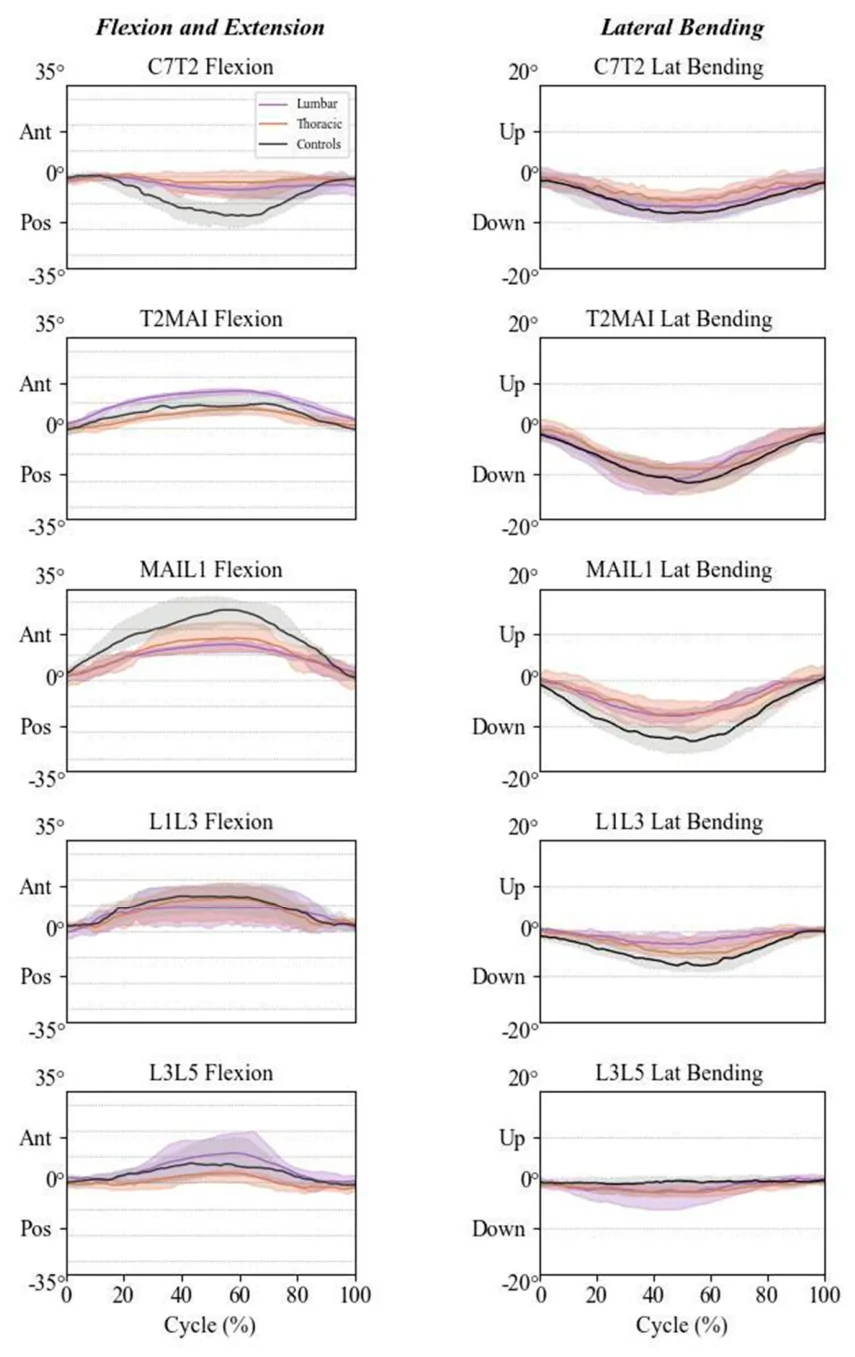
Kinematic curves during the elementary movements (normalized 0–100) in the two corresponding anatomical planes. Relative rotations of the spine line segments are shown. The plots display the median and 25th–75th percentile of spine line segment rotations for the three groups: lumbar (purple), thoracic (orange), and control (gray).

During elementary flexion–extension (Figure 5, left column), two spinal segments showed marked deviations from the control pattern. Specifically, the C7T2 and MAIL1 segments exhibited reductions in the ROM compared to controls. The T2MAI segment showed a greater ROM in the lumbar group than in the thoracic and control populations. At the L1L3 and L3L5 levels, both patient groups demonstrated a general trend comparable to controls, although the lumbar group displayed wider variability, as reflected by broader confidence bands. In addition to segmental mobility, the lumbo-pelvic ratio was analyzed during flexion–extension. The lumbar tumor group exhibited an average LPR of 0.6 (0.5–0.6), while the thoracic tumor group showed an LPR equal to 0.8 (0.5–1.0). Conversely, the control group demonstrated an LPR of 0.5 (0.4–0.7).

### 3.4. Elementary Movements: Lateral Bending

During elementary lateral bending (Figure 3, central column), the pelvic obliquity and thorax lateral bending in the pelvis frame exhibited a smaller ROM in the patient than in the control population, at 4.2° (lumbar group) and 5.3° (thoracic group) vs. 6.8° (control), and 26.8° (lumbar group) and 24.0° (thoracic group) vs. 31.2° (control), respectively.

In this elementary movement, the ROM of the spine segment MAIL1 and L1L3 (Figure 5, right column) were lower in patients than the control population, at 8.1° (lumbar group) and 8.6° (thoracic group) vs. 13.9° (control), and 3.4° (lumbar group) and 5.4° (thoracic group) vs. 7.6° (control), respectively. The ROM of the other spine segments was similar between the patient and control populations.

### 3.5. Elementary Movements: Internal and External Axial Rotations

During elementary internal and external rotations of the trunk (Figure 4, right column), the ROM of pelvic rotation was greater in the patient than in the control population, particularly in the lumbar group, where the ROM was 33.5° vs. 17.3°. Thorax rotation in the pelvis frame and shoulder rotations were smaller in the thoracic group than in the control population, at 13.1° vs. 18.2° and 3.7° vs. 7.0°, respectively.

### 3.6. Patient-Reported Outcomes

Patient-reported outcomes (VAS, ODI, FES-I), collected before the biomechanical analysis, are reported in Table 4. The range of values was quite homogeneous within the patient population, and the mean values indicated a moderate pain level, a moderate fear of falling, and minimal disability.

## 4. Discussion

The present study aimed to evaluate the feasibility, repeatability, and potential clinical applicability of a stereophotogrammetry-based multi-segmental experimental protocol for comprehensive quantitative assessment of trunk motion, thus including the thorax, shoulder, and spine. Building upon the marker-set by Leardini et al. [15], the present protocol was implemented to capture possible coordinated movement across multiple spinal levels through non-invasive skin markers. The analysis was applied to a very rare population of patients affected by primary or metastatic spinal tumors scheduled for en bloc vertebrectomy, divided into two groups according to the location of the tumor. This population was deliberately chosen as a challenging case study to test the robustness of the protocol under critical conditions characterized by altered postural control, local stiffness, and pain. The patient population was compared with age-, sex-, and BMI-matched healthy controls. The primary aim of this study was to verify whether meaningful and repeatable motion patterns could be identified.

The intra-subject repeatability analysis was based on only one representative patient and one healthy subject: it reported good results, as both populations exhibited RMSE values below 10% of the corresponding ROM, and ICC values exceeding 0.8° for all body segments analyzed in this study, i.e., those of the lower limbs, pelvis, thorax, and also spine and shoulder line segments. However, this analysis represents only a preliminary within-session repeatability assessment in two individuals, without establishing repeatability across the entire patient and control cohorts.

Inter-subject reliability was, overall, good to excellent in both groups, indicating that the proposed measurements are able to discriminate between individuals. Slightly lower ICC values observed for thoracic rotation, particularly in patients, likely reflect greater inter-individual variability associated with pathological movement patterns and compensatory motor strategies.

During gait, spatiotemporal parameters (Table 2) showed that these patients, compared with the control group, walked more slowly, with shorter strides and longer stance phases, which was somehow consistent with a cautious gait strategy to maintain stability and to reduce pain or mechanical stress on affected spinal segments, as reported in the literature [22,23,24]. These considerations are in line with previous findings in other spinal pathologies, such as degenerative or stenotic conditions, where reduced walking speed and altered stride parameters reflect compensatory mechanisms for postural control [25,26]. In the present patient population, lower-limb kinematics were found to be physiological, showing preserved walking ability despite the clinical conditions. A controlled pace was not imposed to guarantee a natural self-selected way of walking. Unphysiological kinematics patterns were observed starting from the pelvis, where reduced obliquity and axial rotation were found, reflecting trunk stiffness or motion protection strategies [27]. At the thorax and shoulder levels, kinematic curves of the patient population were comparable to those of the control, but with a smaller ROM, indicating, as expected, a decreased thorax contribution to trunk motion, possibly due to pain avoidance, muscle guarding, or local rigidity from tumor involvement, as deduced also in other populations [28]. The reduction in thorax ROM with respect to that of the pelvis (Figure 2) suggests altered mechanical coupling between the pelvis and thorax, reflecting altered spinal motion, as discussed in the literature [29]. Analysis of the spine line segments revealed comparable mobility between the two populations in the sagittal plane, but this was smaller in the frontal plane in the patients (Table 3).

During the elementary trunk movements, only the rotations corresponding to the primary anatomical plane were reported. Apparently, the present experimental protocol was thus able to isolate the dominant kinematic components, showing small relevant compensatory motion in the other two anatomical planes. Both the thoracic and lumbar patient groups exhibited smaller ROMs than controls in the primary plane, especially during flexion–extension and lateral bending trunk movements. The most pronounced reductions were observed at C7T2 and MAIL1 during flexion–extension. For the former, this was likely influenced by head motion. For the latter, the reduced motion may reflect either local inflammation, mechanical constraints due to tumor mass, or a compensatory limitation to protect the affected area. Axial rotation elementary movement revealed a larger contribution of the pelvis to achieve global trunk axial rotation, likely to limit motion at the spine. This interpretation is further supported by the altered lumbo-pelvic ratio (LPR), which indicates a redistribution of motion between the lumbar spine and pelvis. Together, these findings provide additional insight into movement adaptations in patients with spinal tumors, while the potential clinical implications for postoperative rehabilitation remain to be investigated in future longitudinal studies.

Moreover, pain and functional ability were assessed before the biomechanical analysis by patient-reported outcomes. In particular, we used the VAS scale for pain, the Oswestry Disability Index (ODI) for functional ability, and the Fall Efficacy Scale (FES-I) score, which measures the fear of falling, self-efficacy, and balance confidence. The range of values turned out to be quite homogeneous within the patient population, and they indicated moderate pain levels, moderate fear of falling, and minimal disability grade. We believe that these values should not have a significant impact on the biomechanical analyses.

The results of this study should be interpreted together with its limitations. First of all, kinematic analyses based on skin markers are inherently susceptible to soft tissue artifacts, and the reported patterns also reflect the overall movement of these markers rather than true skeletal motion. Furthermore, knowing that the spine is composed of multiple joints, and that the distribution of the overall trunk motion across each individual joint is cumbersome, the present five spine line segments represent a reasonable compromise compared to existing protocols. Furthermore, the use of biplanar line segments for the shoulders and the spinal region prevents the assessment of out-of-plane rotations, limiting the description of 3D motion coupling mechanisms. Despite these critical aspects, the characteristic movement patterns observed in the two populations provided a reference for comparison, and future developments could include more advanced marker-based or imaging techniques to better capture segmental spinal motion and to enhance anatomical fidelity [30].

Moreover, the study population was small and heterogeneous. However, these are structural limitations because each patient is a unique clinical case. Another potential source of variability for the present results is the walking speed, which was very different between the patient and control populations; this is known to likely affect several kinematic parameters and to diminish the disease-specific biomechanical characteristics. Finaly, the design of the study did not consider the progression speed in order to not alter the natural self-selected way of walking.

This study supports the feasibility of a stereophotogrammetry-based multi-segment trunk motion analysis protocol in patients with primary or metastatic spinal tumors scheduled for en bloc vertebrectomy. Despite the rarity and complexity of these conditions, the protocol identified functional alterations, including reduced spinal mobility, particularly in the sagittal plane, and slower walking speed, relevant measures that are not captured by conventional medical imaging alone.

## 5. Conclusions

The findings of this study support the integration of a quantitative functional assessment into the pre-operative evaluation of patients undergoing complex spinal oncological surgery. By providing descriptive pre-operative information on movement impairments and possible compensatory strategies, this approach may complement conventional clinical and imaging assessments. Larger prospective studies with postoperative longitudinal follow-up are required to determine its potential clinical value in improving surgical planning, postoperative monitoring, and rehabilitation.

## Figures and Tables

**Table 1:** Some demographic data of the 10 patients, together with location and type of spinal tumor; the two patient groups are lumbar (L) and thoracic (T).

N°	ID	Gender	Age (years)	Weight (kg)	Height (cm)	BMI	Type of Tumor
1	TS0	F	34	58	160	22.7	Giant cell tumor of bone (T10)
2	AR0	F	21	70	154	29.5	Giant cell tumor of bone (T11)
3	GA0	M	71	84	183	25.1	Chondrosarcoma (L4)
4	NR0	M	61	100	182	30.2	Chordoma (L4)
5	MR0	M	56	88	176	28.4	Renal metastases (L2)
6	FVP0	M	44	132	182	34.1	Giant cell tumor of bone (L2)
7	RG0	M	43	84	183	26.0	Giant cell tumor of bone (T12)
8	MRO0	M	68	87	172	29.4	Relapsed chondrosarcoma (T7)
9	LS0	M	28	88	170	30.7	Chondrosarcoma (L4)
10	LL0	F	57	72	170	24.9	Metastasis from phylloid cystosarcoma of the breast (T12)
	**Mean**		48.3	86.3	173.2	28.1	
	**St. Dev.**		17.0	19.9	10.1	3.4	

**Table 2:** Spatiotemporal parameters, as median and 25th–75th percentiles.

	Patients	Control
**Cadence (steps/min)**	53.1 (50.0–55.6)	55.3 (51.5–58.3)
**Cycle time (s)**	1.1 (1.1–1.2)	1.1 (1.0–1.2)
**Speed (cm/s)**	99.5 (91.0–105.7)	120.9 (113.1–132.2)
**Speed normalized (% height)**	54.7 (50.5–62.2)	70.6 (62.9–77.6)
**Stride Length**	113.9 (105.9–121.9)	131.8 (117.1–141.6)
**Stride Length (% height)**	65.7 (59.4–69.1)	74.2 (71.5–78.1)
**Stance (% gait cycle)**	62.3 (61.6–62.8)	60.6 (59.9–61.7)
**Swing (% gait cycle)**	37.7 (37.2–38.4)	39.4 (38.3–40.1)

**Table 3:** ROM (in degrees) of the five-line segments in the sagittal (top five rows) and frontal (bottom five rows) planes during gait. The median and 25th–75th percentile (in brackets) are reported.

	Patients	Controls
**C7T2 Sagittal**	2.1 (1.4–2.9)	1.8 (1.4–2.5)
**T2MAI Sagittal**	1.7 (1.4–2.1)	2.0 (1.4–2.9)
**MAIL1 Sagittal**	3.8 (2.7–4.9)	4.3 (3.4–6.5)
**L1L3 Sagittal**	4.9 (3.9–6.7)	5.2 (4.1–8.0)
**L3L5 Sagittal**	3.6 (2.6–4.2)	3.5 (2.5–5.3)
**C7T2 Frontal**	5.3 (4.2–7.9)	8.7 (6.2–11.4)
**T2MAI Frontal**	6.0 (4.6–7.2)	9.7 (7.9–11.1)
**MAIL1 Frontal**	3.9 (3.0–5.1)	6.0 (4.7–7.1)
**L1L3 Frontal**	3.0 (2.5–4.6)	5.6 (4.6–7.1)
**L3L5 Frontal**	3.6 (2.7–5.1)	6.1 (4.8–7.7)

**Table 4:** Pain and functional assessment by patient-reported outcomes.

Patient Number	Pain Pre-Op	Fes-I Pre-Op	ODI Pre-Op
1	6	29	21 (42%)
2	2	28	22 (44%)
3	4	32	16 (32%)
4	2	23	15 (30%)
5	2	31	22 (44%)
6	4	23	17 (34%)
7	0	20	1 (2%)
8	4	19	12 (24%)
9	6	21	14 (28%)
10	6	33	24 (48%)
Mean	3.6	25.9	16.4
St.Dev.	2.1	5.3	6.7

## Data Availability

The raw data supporting the conclusions of this article will be made available by the corresponding author on request.

## Supplementary Materials

The following supporting information can be downloaded at https://www.mdpi.com/article/10.3390/cancers18162599/s1, Table S1: Inter-subject reliability of kinematic variables in patients. Intra-class correlation coefficient (ICC(3,k)), ninety-five percent confidence intervals (95% CI), standard error of measurement (SEM). S1A: Level walking; S1B: flexion–extension; S1C: lateral bending; S1D: axial rotation.
