# Supplementary material for: Quantitative Functional Assessment of Patients Undergoing En Bloc Vertebrectomy for Spinal Tumors: A Multi-Segment Motion Analysis Study
Source: Cancers (Basel). 2026 Aug 12;18(16):2599. doi: 10.3390/cancers18162599 (PMC13510479; doi:10.3390/cancers18162599)
Supplement: Supplementary file 1 [file cancers-18-02599-s001.zip › cancers-4413999-supplementary.pdf]

Table S1. Inter-subject reliability of kinematic variables in patients. Intraclass correlation coefficient (ICC(3,k)), ninety-five percent confidence intervals (95% CI) standard error of measurement (SEM). **S1A: Level Walking; S1B: Flexion Extension; S1C: Lateral Bending; S1D: Axial Rotation**

S1A. Level Walking

	Controls		Patients	
Variables	ICC (3, k)	SEM	ICC (3, k)	SEM
Tho Flexion (LAB r.f.)	0.76 (0.40–0.94)	1.7	0.63 (0.42–0.93)	1.4
Tho Bending (LAB r.f.)	0.96 (0.88–0.99)	2.7	0.95 (0.86–0.98)	2.4
Tho Rotation (LAB r.f.)	0.94 (0.71–0.98)	2.6	0.50 (0.40–0.81)	2.3
Tho Flexion (PEL r.f.)	0.93 (0.83–0.99)	2.0	0.84 (0.67–0.96)	1.3
Tho Bending (PEL r.f.)	0.94 (0.82–0.99)	4.2	0.98 (0.94–0.99)	3.3
Tho Rotation (PEL r.f.)	0.60 (0.42–0.88)	3.6	0.96 (0.89–0.99)	2.3
C7T2 Flexion	0.79 (0.61- 0.96)	1.4	0.81 (0.73–0.96)	1.7
T2MAI Flexion	0.78 (0.67–0.96)	1.5	0.84 (0.55–0.96)	0.97
MAIL1 Flexion	0.65 (0.51–0.90)	3.6	0.96 (0.88–0.99)	2.3
L1L3 Flexion	0.83 (0.58–0.95)	3.6	0.94 (0.83–0.99)	2.6
L3L5 Flexion	0.84 (0.71–0.92)	2.7	0.88 (0.61–0.98)	1.4
C7T2 Lat Bending	0.97 (0.93–0.99)	3.6	0.94 (0.83–0.99)	2.7
T2MAI Lat Bending	0.96 (0.91–0.99)	2.6	0.98 (0.92–0.99)	2.4
MAIL1 Lat Bending	0.81 (0.73–0.92)	3.0	0.96 (0.89–0.98)	1.7
L1L3 Lat Bending	0.95 (0.83–0.99)	2.8	0.98 (0.96–0.99)	2.0
L3L5 Lat Bending	0.95 (0.81–0.99)	2.5	0.95 (0.87–0.99)	2.0

S1B. Flexion Extension

	Controls		Patients	
Variables	ICC (3, k)	SEM	ICC (3, k)	SEM
Tho Flexion (LAB r.f.)	0.98 (0.65–0.99)	10.2	0.99 (0.98–0.99)	15.3
Tho Bending (LAB r.f.)	0.73 (0.65–0.92)	2.4	0.92 (0.78–0.97)	1.9
Tho Rotation (LAB r.f.)	0.76 (0.65–0.94)	2.8	0.50 (0.43–0.86)	3.4
Tho Flexion (PEL r.f.)	0.97 (0.67–0.99)	9.1	0.97 (0.93–0.99)	13.4
Tho Bending (PEL r.f.)	0.90 (0.95–0.97)	3.0	0.85 (0.60–0.95)	1.6
Tho Rotation (PEL r.f.)	0.88 (0.65–0.98)	2.8	0.81 (0.44–0.95)	2.5
C7T2 Flexion	0.84 (0.66–0.96)	9.4	0.79 (0.68–0.94)	4.6
T2MAI Flexion	0.92 (0.66–0.98)	5.4	0.93 (0.81–0.98)	4.4
MAIL1 Flexion	0.95 (0.66–0.99)	6.4	0.97 (0.92–0.99)	7.4
L1L3 Flexion	0.94 (0.66–0.98)	5.1	0.97 (0.93–0.99)	8.0
L3L5 Flexion	0.96 (0.65–0.99)	7.5	0.89 (0.68–0.97)	6.0
C7T2 Lat Bending	0.96 (0.67–0.99)	0.7	0.96 (0.67–0.99)	0.7
T2MAI Lat Bending	0.93 (0.67–0.98)	2.4	0.92 (0.78–0.98)	3.7
MAIL1 Lat Bending	0.93 (0.89–0.98)	1.7	0.97 (0.92–0.99)	3.0
L1L3 Lat Bending	0.78 (0.66–0.94)	2.8	0.84 (0.56–0.95)	2.9
L3L5 Lat Bending	0.83 (0.66–0.95)	2.2	0.76 (0.66–0.93)	2.7

S1C. Lateral Bending

	Controls		Patients	
Variables	ICC(3, k)	SEM	ICC(3, k)	SEM
Tho Flexion (LAB r.f.)	0.89 (0.63–0.97)	4.7	0.97 (0.94–0.93)	5.8
Tho Bending (LAB r.f.)	0.98 (0.93–0.99)	7.8	0.98 (0.92–0.93)	6.8
Tho Rotation (LAB r.f.)	0.92 (0.77–0.98)	5.9	0.96 (0.88–0.98)	5.7
Tho Flexion (PEL r.f.)	0.95 (0.82–0.98)	5.1	0.97 (0.94–0.99)	5.4
Tho Bending (PEL r.f.)	0.99 (0.96–0.99)	6.9	0.98 (0.94- 0.99)	5.7
Tho Rotation (PEL r.f.)	0.96 (0.85–0.99)	4.2	0.96 (0.88–0.99)	3.3
C7T2 Flexion	0.93 (0.75- 0.98)	2.8	0.93 (0.71–0.97)	2.4
T2MAI Flexion	0.81 (0.62–0.93)	2.2	0.93 (0.80–0.98)	2.0
MAIL1 Flexion	0.92 (0.60–0.96)	3.5	0.96 (0.74–0.99)	2.9
L1L3 Flexion	0.88 (0.65–0.97)	3.3	0.98 (0.68–0.99)	2.8
L3L5 Flexion	0.87 (0.65–0.97)	2.3	0.93 (0.78–0.98)	2.1
C7T2 Lat Bending	0.93 (0.76–0.98)	3.4	0.90 (0.65–0.95)	3.4
T2MAI Lat Bending	0.94 (0.74–0.97)	4.3	0.94 (0.81–0.99)	3.6
MAIL1 Lat Bending	0.98 (0.90–0.99)	4.6	0.98 (0.96–0.99)	3.4
L1L3 Lat Bending	0.91 (0.72–0.97)	2.8	0.94 (0.78–0.98)	2.1
L3L5 Lat Bending	0.94 (0.81–0.99)	2.2	0.98 (0.96–0.99)	3.0

S1D. Axial Rotation

	Controls		Patients	
Variables	ICC(3, k)	SEM	ICC(3, k)	SEM
Tho Flexion (LAB r.f.)	0.87 (0.67–0.96)	1.0	0.72 (0.40–0.92)	1.3
Tho Bending (LAB r.f.)	0.94 (0.76–0.98)	1.7	0.80 (0.69–0.92)	1.1
Tho Rotation (LAB r.f.)	0.96 (0.87–0.98)	3.3	0.84 (0.81–0.98)	2.8
Tho Flexion (PEL r.f.)	0.97 (0.90–0.99)	0.7	0.75 (0.44–0.93)	1.2
Tho Bending (PEL r.f.)	0.93 (0.76–0.99)	1.1	0.61 (0.21–0.88)	1.1
Tho Rotation (PEL r.f.)	0.94 (0.82–0.99)	1.6	0.90 (0.71–0.97)	2.4
